# Predictors of mistimed, and unwanted pregnancies among women of childbearing age in Rufiji, Kilombero, and Ulanga districts of Tanzania

**DOI:** 10.1186/1742-4755-11-63

**Published:** 2014-08-08

**Authors:** Amon Exavery, Almamy Malick Kanté, Mustafa Njozi, Kassimu Tani, Henry V Doctor, Ahmed Hingora, James F Phillips

**Affiliations:** 1 Ifakara Health Institute, Plot 463, Kiko Avenue, Mikocheni, P.O Box 78373, Dar es Salaam, Tanzania; 2 Department of Population and Family Health, Mailman School of Public Health, Columbia University, New York, USA; 3 Jhpiego, Dar es Salaam, Tanzania; 4 United Nations Office on Drugs and Crime, United Nations House, Abuja, Nigeria

**Keywords:** Mistimed pregnancy, Unwanted pregnancy, Women, Characteristics, Rufiji, Kilombero, Ulanga, Tanzania

## Abstract

**Background:**

While unintended pregnancies pose a serious threat to the health and well-being of families globally, characteristics of Tanzanian women who conceive unintentionally are rarely documented. This analysis identifies factors associated with unintended pregnancies—both mistimed and unwanted—in three rural districts of Tanzania.

**Methods:**

A cross-sectional survey of 2,183 random households was conducted in three Tanzanian districts of Rufiji, Kilombero, and Ulanga in 2011 to assess women’s health behavior and service utilization patterns. These households produced 3,127 women age 15+ years from which 2,199 gravid women aged 15–49 were selected for the current analysis. Unintended pregnancies were identified as either mistimed (wanted later) or unwanted (not wanted at all). Correlates of mistimed, and unwanted pregnancies were identified through Chi-squared tests to assess associations and multinomial logistic regression for multivariate analysis.

**Results:**

Mean age of the participants was 32.1 years. While 54.1% of the participants reported that their most recent pregnancy was intended, 32.5% indicated their most recent pregnancy as mistimed and 13.4% as unwanted. Multivariate analysis revealed that young age (<20 years), and single marital status were significant predictors of both mistimed and unwanted pregnancies. Lack of inter-partner communication about family planning increased the risk of mistimed pregnancy significantly, and multi-gravidity was shown to significantly increase the risk of unwanted pregnancy.

**Conclusions:**

About one half of women in Rufiji, Kilombero, and Ulanga districts of Tanzania conceive unintentionally. Women, especially the most vulnerable should be empowered to avoid pregnancy at their own will and discretion.

## Background

Unintended pregnancies, defined as pregnancies that are mistimed or unwanted, pose significant public health concern especially in low- and middle-income countries due to their association with adverse health, social, and economic outcomes [1-5]. An obvious consequence of unintended pregnancy is induced abortion, which is often unsafe in countries where the practice is illegal [3,6]. Adverse pregnancy outcomes attributable to unintended pregnancy such as low birth weight, premature birth [1], maternal depression [7-9], anxiety [10], poor psychological wellbeing [11], and poor utilization of antenatal and delivery care [12,13] have been documented. Further documentation of the serious consequences of unintended pregnancy exists from studies within other low- and middle-income countries [12-15].

In 2008, an estimated 208 million pregnancies occurred worldwide, out of which 86 million (41%) were unintended. Of these (unintended pregnancies), 33 million (39%) ended in unintended births, 41 million (48%) in abortions, and 11 million (13%) in miscarriages [2]. The 2010 Tanzania Demographic and Health Survey (TDHS) estimated that 26% of births that occurred five years preceding the survey were unintended (22% were mistimed; 4% were unwanted), and these estimates showed no significant change from those observed during the 2004–05 TDHS [16]. Unintended pregnancies contribute significantly to unwanted population growth, which consequently compromises provision of adequate social services [17]. Therefore, elimination of unintended pregnancy is important not only to reduce fertility and the rate of unwanted population growth, but also to enhance the wellbeing of women and their families [18].

Unintended pregnancies are primarily caused by nonuse and/or failure of contraceptives [17], implying that correct and consistent use of effective contraceptives can lead to prevention of unintended pregnancies [19]. Results of a paper published in 2013 from a cross-sectional survey conducted in Mwanza, Tanzania, among young adults aged 15–30 years found that older age, lower educational level, unmarried status, lack of knowledge of where to access condoms, increased number of sexual partners, and younger age at sexual debut were significantly associated with unintended pregnancy [19]. In this study, the prevalence of unintended pregnancy was estimated at 30.5% among women who had never been married and 23.7% among women who had ever been married. A community-based survey in Nigeria found that 28.0% of women had experienced an unwanted pregnancy at some point in their lives. Risk factors identified by the study included marital status, parity, place of residence, religion, and socio-economic status [6]. Similarly, a matched case–control study in India reported religion, and socio-economic status (measured by wealth index) as significant predictors of unwanted pregnancy [1].

Additional predictors of unwanted pregnancy reported from this study were partners’ education of at least high school level and female sex of the most recent child [1]. Unfortunately, behavioral interventions that have attempted to reduce unintended pregnancies have shown no evidence of success.

For example, a cluster randomized trial (CRT) in Tanzania that was conducted to evaluate the *MEMA kwa Vijana* intervention in Mwanza showed no effect on self-reported unintended pregnancy at any point since its introduction [20,21]. Also an evaluation of the *Stepping Stone* intervention in South Africa found that women in the intervention arm were 1.45 times more likely than their control counterparts to report unintended pregnancy after 24 months, although the 45% increase was not statistically significant (p = 0.110) [22]. Similarly, the Regai Dzive Shiri project in Zimbabwe showed no effect on unintended pregnancy among young women [23].

Much is still unknown about the risk factors for unintended or unplanned pregnancy. Also, previous studies have assessed risk factors for unintended pregnancy without unpacking items that actually make up unintended pregnancy and what affects each item.

The literature defines unintended pregnancy within two categories—mistimed (the pregnancy is desired, but at a later time), or unwanted (the pregnancy is not desired at all) [16,24]. While these are often grouped together as unintended pregnancies, there may be differences in the risk factors for mistimed pregnancies versus unwanted pregnancies. Women experiencing mistimed pregnancies likely feel different towards the pregnancy as women experiencing entirely unwanted pregnancies. Therefore, identification of the characteristics associated with mistimed and unwanted pregnancies is vital to understand vulnerable groups and subsequently to inform programs about necessary components for effective interventions. Accordingly, the objectives of this study are (1) to estimate the magnitude of mistimed pregnancy, and unwanted pregnancy, and (2) to identify factors associated with mistimed pregnancy and unwanted pregnancy among women of reproductive age in Rufiji, Kilombero, and Ulanga districts of Tanzania.

## Methods

### Data source, study area, and study population

Data were collected through a cross-sectional household survey conducted in 2011 in Rufiji, Kilombero, and Ulanga districts of Tanzania. Women aged 15 to 49 years (in some cases, above 49 years) were surveyed to assess health behavior and service utilization patterns for women and their children under five years old. Women over age 49 were interviewed only if they were responsible for total caretaking of a child under age five. These data served as the baseline against which progress was measured after the introduction of a cadre of community health workers through a cluster randomized trial. Among other health improvement goals, this intervention was aimed at accelerating achievement of the Millenium Development Goals (MDG) 4 and 5 which are focused on improving child and maternal health, respectively. While details regarding the cluster randomized trial [25] and the baseline survey itself [26] are further described elsewhere, 2,199 gravid women aged 15 to 49 years who provided information about the intentionality of their recent pregnancies were selected for the analyses reported herein.

### Variables and definitions

The outcome of interest analyzed in this study was pregnancy intentions and was operationalized within three categories as intended, mistimed, and unwanted. Women responded to a survey question asking them to reflect about whether their most recent pregnancy was intended at the time of conception, intended but at a later time, or entirely unintended. Therefore a pregnancy was defined as “wanted” or “intended” if the respondent reported that she wanted to become pregnant at the time of conception. If the woman reported that she wanted the pregnancy but at a later time, her pregnancy was defined as “mistimed”. Finally, if the woman reported that she did not want to become pregnant at all, the pregnancy was defined as “unwanted”. These definitions are standard and have been applied in other studies [16,24].

Based on the literature reviewed earlier, several independent variables were included in the current analyses. This included age in years (<20, 20–34, and >34), marital status (married, ever married, and single), formal education (none, primary, and secondary/higher), religion (Christianity, Islam, and Others), ethnicity (Pogoro, Ndengereko, Ngindo, Ndamba, Ngoni, Sukuma, Hehe, Bena, and Others), district of residence (Kilombero, Rufiji, and Ulanga), type of residence (rural, and urban), and socio-economic status (poor, middle, and rich). Other independent variables included were inter-partner discussion about family planning (yes, and no), contraceptive use (not using, ever used, and currently using), and gravidity (one, two-four, and five or more). Gravidity was used as a proxy for fertility. Socio-economic status was constructed using principal component analysis (PCA) of household assets owned. The PCA included ownership of a toilet, type of the toilet, and source of drinking water. Other assets such as bicycle, radio, refridgerator, etc. were not available for the PCA.

### Data analysis

Data management and analysis were performed using STATA (version 11) statistical software. For descriptive statistics, frequency distribution of respondents across categories of the variables was initially conducted. Analysis of continuous variables such as age involved calculation and presentation of summary statistics including mean and standard deviation. Cross tabulations or bivariate analysis of pregnancy intentions by each of the independent variables was performed. The degree of association between each pair of cross-tabulated categorical variables was tested using Pearson’s Chi Square (*χ*^2^) test. Finally, multivariate analysis was performed using multinomial logistic regression with the “intended” group as the baseline outcome. To achieve parsimoniousness, a variable was retained in the model if the log likelihood ratio test showed that its presence improved the overall model. The level of significance at which a variable was identified as a predictor of the outcome was set at 5%. Both relative risk ratios (RRR) and their corresponding 95% confidence intervals (CI) were presented.

### Ethical approval

This analysis is derived from a primary study that received ethical clearances from the Medical Research Coordination Committee (MRCC) of the National Institute for Medical Research (NIMR) in Tanzania, the Ifakara Health Institute’s Institutional Review Board (IRB) and Columbia University. During field data collection, participation in the study was voluntary with respondents signing a written consent form before the interview. There was no link between the signed consent form and respondent data subsequently. The data remained anonymous throughout data analysis.

## Results

### Profile of respondents

Demographic characteristics of the 2,199 participants are presented in Table 1. All the respondents were gravid women aged 15–49 years with a mean age of 32.1 years (standard deviation = 8.6 years). Slightly more than one half of the respondents (51.6%) were aged 20–34 years. The youngest (<20 years) respondents accounted for 7.1% and the oldest (>34 years) accounted for 41.3% of the total respondents. Majority of the women (79.4%) were married. Furthermore, 73.1% and 5.5% had primary education and secondary/higher education, respectively.

**Table 1:** Sample characteristics of women analyzed in the assessment of factors affecting pregnancy intentions in Kilombero, Rufiji, and Ulanga districts of Tanzania, 2011

**Variable**	**Number of respondents (n)**	**Percent (%)**
**OVERALL**	**2,199**	**100.0**
**Pregnancy intentions**		
Intended	1,189	54.1
Mistimed	715	32.5
Unwanted	295	13.4
**Age (years)**		
<20	157	7.1
20–34	1,135	51.6
>34	907	41.3
Mean = 32.1, SD = 8.6, Min = 15, Max = 49	–	–
**Marital status**		
Married	1,745	79.4
Ever married	201	9.1
Single	253	11.5
**Ethnicity**^**‡**^		
Pogoro	351	16.0
Ndengereko	312	14.2
Ngindo	311	14.2
Ndamba	203	9.2
Ngoni	176	8.0
Sukuma	158	7.2
Hehe	149	6.8
Bena	111	5.1
Others	427	19.4
**Education**		
Never been to school	472	21.5
Primary	1,607	73.1
Secondary/higher	120	5.5
**Gravidity**		
1	357	16.2
2–4	990	45.0
>4	852	38.7
**Religion**		
Christian	1,014	46.1
Muslim	1,114	50.7
Traditional/other	71	3.2
**Socio-economic status**^**‡**^		
Poor	711	34.5
Middle	709	34.4
Rich	642	31.1
**District**		
Kilombero	1,399	63.6
Rufiji	471	21.4
Ulanga	329	15.0
**Type of residence**^**‡**^		
Rural	1,787	81.6
Urban	404	18.4

^‡^Some missing data (n < 2,199).

The majority of participants (81.6%) resided in rural areas while the rest (18.4%) resided in urban or semi-urban settings. Kilombero district had 63.6% of the total respondents, while Rufiji district had 21.4%, and Ulanga district had 15.0% of the respondents. Almost one half (50.7%) of the respondents were Muslims, 46.1% were Christians, and 3.2% were of traditional or other unspecified beliefs.

### Pregnancy intentions by maternal characteristics

Potential predictors of pregnancy intentions were first analyzed descriptively using bivariate analysis. The results of this analysis are presented in Table 2. Overall, slightly more than one half (54.1%) of the respondents reported that their last pregnancies were intended, whereas 32.5% and 13.4% reported that their last pregnancies were mistimed and unwanted, respectively. These proportions varied significantly by some maternal characteristics. While the proportion of intended pregnancies was lowest at 38.9% among the youngest women (age <20 years) and highest at 56.5% among the oldest women, mistimed pregnancies were highest (44.0%) among the youngest (age <20 years) women and lowest (27.7%) among the oldest (age >34 years) women. Unwanted pregnancies were 17.2% among the youngest, 10.9% among the 20–34 year-olds, and 15.9% among the oldest women. These differences in the pregnancy intentions by age group were statistically significant (P < 0.001). The association between marital status and pregnancy intentions was statistically significant (P < 0.001). The proportion of intended pregnancies was highest at 57.1% among married women, declined slightly to 56.7% among ever-married women and became lowest at 30.8% among single women. In contrast, the proportions of both mistimed and unwanted pregnancies were lowest among married women and highest among single women.

**Table 2:** Percent distribution of pregnancy intentions by women’s characteristics in the assessment of factors affecting pregnancy intentions in Rufiji, Kilombero, and Ulanga districts of Tanzania, 2011

**Variable**	**Number of women**	**Pregnancy Intentions**			**P-value**
		**Intended**	**Mistimed**	**Unwanted**	
**OVERALL**	**2,199**	**54.1**	**32.5**	**13.4**	**–**
**Age (years)**					<0.001
<20	157	38.9	44.0	17.2	
20–34	1,135	54.3	34.8	10.9	
>34	907	56.5	27.7	15.9	
**Marital status**					<0.001
Married	1,745	57.1	31.2	11.7	
Ever married	201	56.7	28.4	14.9	
Single	253	30.8	45.1	24.1	
**Ethnicity**					0.317
Pogoro	351	49.6	34.8	15.7	
Ndengereko	312	54.2	33.0	12.8	
Ngindo	311	54.7	35.7	9.7	
Ndamba	203	52.7	31.0	16.3	
Ngoni	176	48.3	34.7	17.1	
Sukuma	158	60.1	27.9	12.0	
Hehe	149	52.4	34.2	13.4	
Bena	111	58.6	30.6	10.8	
Others	427	57.6	29.3	13.1	
**Education**					0.288
Never been to school	472	57.6	28.4	14.0	
Primary	1,607	53.2	33.5	13.3	
Secondary/higher	120	51.7	35.8	12.5	
**Gravidity**					<0.001
1	357	49.6	36.1	14.3	
2–4	990	57.5	32.6	9.9	
>4	852	52.0	30.9	17.1	
**Religion**					0.023
Christian	1,014	52.0	33.1	14.9	
Muslim	1,114	54.9	32.6	12.5	
Traditional/other	71	70.4	22.5	7.0	
**Socio-economic status**					0.369
Poor	711	52.7	33.8	13.5	
Middle	709	54.6	33.2	12.3	
Rich	642	54.1	30.4	15.6	
**District**					<0.001
Kilombero	1,399	53.0	31.7	15.4	
Rufiji	471	54.6	32.3	13.2	
Ulanga	329	58.1	36.5	5.5	
**Type of residence**					0.016
Rural	1,787	54.3	33.2	12.5	
Urban	404	53.5	29.0	17.6	
**Contraceptive use status**					0.020
Not using	707	53.2	31.7	15.1	
Ever used	579	55.6	29.2	15.2	
Currently using	913	53.8	35.3	11.0	
**Inter–partner discussion about family planning**					<0.001
Yes	903	59.7	29.1	11.2	
No	1,296	50.2	34.9	15.0	

Other factors significantly associated with pregnancy intentions as indicated through bivariate analysis were gravidity (P < 0.001), inter-partner discussion about family planning (P < 0.001), district of residence (P < 0.001), religion (P = 0.023), type of residence (P = 0.016), and contraceptive use status (P = 0.020).

Education (P = 0.288) and household socioeconomic status (P = 0.369) were not statistically significantly associated with pregnancy intentions in the bivariate analysis, despite the fact that their significance has been reported in other studies, for example [19] and [6] respectively.

### Multivariate analysis

The results from multivariate analysis of correlates of mistimed, and unwanted pregnancies are displayed in Table 3. Age was significantly associated with mistimed pregnancy yet not with unwanted pregnancy. Participants aged 20–34 experienced a 47% lower risk of mistimed pregnancy compared to those in age group <20 years (RRR = 0.53, 95% CI 0.35–0.83). Participants over 34 years of age experienced a 65% lower risk of mistimed pregnancy compared to those <20 years old (RRR = 0.35, 95% CI 0.22–0.57).

**Table 3:** Multivariate multinomial logistic regression of factors associated with mistimed and unwanted pregnancies among women in three districts of Tanzania, 2011 (n = 2,054)

	**Mistimed pregnancy**		**Unwanted pregnancy**	
**Variable**	**RRR**	**95%Confidence Interval (CI)**	**RRR**	**95%Confidence Interval (CI)**
**Age (years)**				
<20 (ref)	1.00	–	1.00	–
20–34	0.53**	0.35–0.83	0.63	0.34–1.17
>34	0.35***	0.22–0.57	0.60	0.31–1.18
**Marital status**				
Married (ref)	1.00	–	1.00	–
Ever married	0.93	0.66–1.32	1.14	0.72–1.81
Single	2.24***	1.58–3.17	4.63***	2.99–7.15
**Gravidity**				
1 (ref)	1.00	–	1.00	–
2–4	1.18	0.85–1.64	0.98	0.62–1.57
>4	1.76**	1.19–2.61	2.65***	1.54–4.54
**Religion**				
Christian (ref)	1.00	–	1.00	–
Muslim	0.85	0.68–1.06	0.71**	0.52–0.97
Traditional/other	0.44**	0.20–0.97	0.51	0.14–1.84
**District**				
Kilombero (ref)	1.00	–	1.00	–
Rufiji	0.96	0.70–1.31	0.78	0.51–1.20
Ulanga	1.15	0.85–1.55	0.42**	0.24–0.72
**Contraceptive use**				
Not using (ref)	1.00	–	1.00	–
Ever used	1.11	0.87–1.42	0.81	0.58–1.15
Currently using	0.93	0.70–1.22	1.00	0.70–1.41
**Inter–partner discussion about family planning**				
Yes (ref)	1.00	–	1.00	–
No	1.49***	1.20–1.84	1.48**	1.10–1.99
**Education**				
Never been to school (ref)	1.00	–	1.00	–
Primary	1.14	0.87–1.49	0.83	0.58–1.18
Secondary/higher	1.24	0.76–2.03	0.70	0.35–1.42
**Socio-economic status**				
Poor (ref)	1.00	–	1.00	–
Middle	0.88	0.68–1.14	0.92	0.64–1.32
Rich	0.85	0.64–1.13	1.02	0.69–1.49
**Type of residence**				
Rural (ref)	1.00	–	1.00	–
Urban	1.09	0.83–1.45	0.77	0.54–1.09

***P < 0.001, **P < 0.05, RRR = Relative risk ratio; CI = Confidence interval; (ref) = Reference category.

Marital status showed a remarkable influence on both mistimed and unwanted pregnancies. Women who were single (unmarried) were 2.24 times more likely than married women to have experienced mistimed pregnancy (RRR = 2.24, 95% CI 1.58–3.17) and 4.63 times more likely than married women to have had an unwanted pregnancy (RRR = 4.63, 95% CI 2.99–7.15). The risk of experiencing a mistimed pregnancy was 1.76 times higher for women who had had four or more pregnancies than for those who had experienced one pregnancy (RRR = 1.76, 95% CI 1.19–2.61). Similarly, the risk of an unwanted pregnancy was 2.65 times higher for those with multi-gravidity of four or more pregnancies than for women who had been pregnant once. (RRR = 2.65, 95% CI 1.54-4.54).

Inter–partner discussion about family planning revealed a remarkable effect on both mistimed and unwanted pregnancies. Women with no inter–partner discussion about family planning were 1.49 (RRR = 1.49, 95% CI 1.20–1.84) times more likely to have a mistimed pregnancy and 1.48 (RRR = 1.48, 95% CI 1.10–1.99) times more likely to have an unwanted pregnancy than those who reported discussing family planning with their partners.

Religion also showed an association with pregnancy intentions. Women with traditional or other unspecified beliefs experienced a 56% less risk of mistimed pregnancy (RRR = 0.44, 95% CI 0.20-0.97) than Christian, while Muslim women experienced 29% lower risk of unwanted pregnancy (RRR = 0.71, 95% CI 0.52-0.97) than Christian. District of residence also had a relationship with pregnancy intentions. The risk of unwanted pregnancy was 58% lower in Ulanga than in Kilombero district (RRR = 0.42, 95% CI 0.24–0.72).

## Discussion

Unintended pregnancies pose significant risks to the health and general welfare of individuals and families. As such, measures aimed at reducing mistimed and unwanted pregnancies should be pursued. Implementation of appropriate interventions to reduce unintended pregnancies requires an understanding of the underlying factors that influence such pregnancies. Under this goal, this analysis examined the magnitude and correlates of unintended pregnancy among women of reproductive age in Kilombero, Rufiji, and Ulanga districts of Tanzania.

Results show that close to one half of the women analyzed had not intended their most recent pregnancies (32.5% mistimed and 13.4% unwanted). Although these figures were higher than the national estimates of 22.0% mistimed and 4.0% unwanted from the 2010 Tanzania DHS [16], in both cases, the proportion of mistimed pregnancies significantly outnumbered the proportion of unwanted pregnancies. The comparatively higher estimates of unintended pregnancies in the study communities over national estimates are partially explained by the increased diversity of contexts accounted for nationally. The 2008–09 Kenya Demographic and Health Survey (KDHS) recorded 43.0% (26.0% mistimed and 17.0% unwanted) of current pregnancies among married women as unintended [27]. This proportion was 45.0% in the 2003 KDHS [28]. These findings from both surveys in Kenya are very similar with those found in our analysis in Kilombero, Rufiji and Ulanga districts of Tanzania. Furthermore, an estimated 46.0% of pregnancies were unintended in East Africa in 2008 (where Tanzania is situated) [2]. This proportion matches the result from our study and is similar to estimates of unintended pregnancies in regions of Asia, Europe, and Latin America and Caribbean [2].

Multivariate analysis revealed several factors associated with both mistimed and unwanted pregnancies, each independent of the other. Marital status was the strongest factor, with single women showing a proclivity for both mistimed and unwanted pregnancies. This suggests, in part, that single women likely engage in sexual activities for motivations other than childbearing such as pleasure, social status, fiscal gain, or other exchanges. Accordingly, researchers have argued that single women are likely to be young, still in school, and consequently more likely to conceive mistakenly as they are not prepared for childbearing [29]. The observed significance of marital status in predicting unintended pregnancies in the study area is consistent with another study conducted in Tanzania [19] and others from elsewhere [30,31].

The study results also showed that mistimed pregnancy was most likely to occur in younger aged women, and showed decreasing likelihood with increasing age. This observation supports similar findings from slum settlements of Nairobi, Kenya [31] and findings from Nigeria [6], but contradicts findings from the Tanzania DHS [16]. As noted previously, many of the younger women are not yet married and are still in school, making them likely to have sexual intercourse for reasons other than childbearing [29]. Therefore, a pregnancy in these situations is likely to be unintended. Additionally, younger women may have lower knowledge and skills surrounding pregnancy control mechanisms such as contraceptive use compared with their older counterparts, thus increasing their likelihood of experiencing unintended pregnancies.

Furthermore, women in relationships where inter-partner discussion about family planning matters was not taking place were significantly more likely than their counterparts to experience unintended pregnancies. Unlike this study, previous studies have ignored inter-partner communication about family planning as a possible predictor of pregnancy intentions, therefore limited comparison of findings. However, extant evidence show that existence of discussions between partners about fertility and family planning-related matters is strongly associated with contraceptive use [32-35]. This supports the earlier assertion that if effective contraceptives are available and correctly used, unintended pregnancies can be prevented. This underscores the need to empower couples to understand the importance of making family planning decisions jointly [36] since this reduces unplanned pregnancies through increased use of contraception.

Religion was shown to have a significant effect on unintended pregnancy, with traditionalists or those with other unspecified beliefs exhibiting less risk for mistimed pregnancy compared to Christian women, and with Muslim women showing less risk of unwanted pregnancy compared to Christian women. While categorization of religions may be arbitrary depending on contexts, the overall significance of religion in predicting unintended pregnancy shown in this study is consistent with others [1,6]. One study in Mwanza, Tanzania, however, treated religion similar to this study, but yet found no significant relationship between religion and unintended pregnancy [19]. In contrast to our study, other studies have found positive association between Muslim religion and unwanted pregnancy, for example in India, Nepal, and Sri Lanka [37,38]. Further assessment of religion in relation to fertility and population growth exist [39], and findings are generally mixed, thus a need for further research to reveal the likely underlying mechanisms of the relationship between them.

Finally, the risk of unwanted pregnancy was significantly lower in Ulanga than in Kilombero district. Although specific explanations for this observation are lacking, the variation observed may be attributed to differential access to information and pregnancy control services as well as cultural beliefs, norms, and practices pertaining to pregnancies. Place of residence has been found in other studies as a significant predictor of unwanted pregnancy, where the risk of unwanted pregnancy differs by location [1,6]. In Nigeria for example, Singh and Hussain found a higher risk of unwanted pregnancy in the North than in the South, and also in the rural than in the urban areas [2]. Similarly, Ikamari *et al.* found in Nairobi that women in non-Slum settlements were more likely than their counterparts in Slum settlements to experience unplanned pregnancy [31]. Therefore place of residence has significant association with pregnancy intentions in the study area.

### Limitations

As respondents were retrospectively asked of their pregnancy intentions, this may have caused recall bias which may consequently have affected the estimated prevalence of unintended pregnancy in the study area. The study was also cross-sectional, a design which limits causal inferences because of their snapshot nature. Generalization of the findings countrywide and/or beyond may also not be guaranteed since three districts only were studied.

## Conclusions

Close to one half of pregnancies among 15–49 year–old women in Rufiji, Kilombero and Ulanga districts of Tanzania are unintended. Overall, factors affecting mistimed, and unwanted pregnancies are very similar, yet not entirely the same. Women at a higher risk of mistimed pregnancies are younger, single, multi-gravidous (>4 pregnancies) women, and those who lack between–partner communication about family planning. Those at a higher risk of unwanted pregnancies are single, multi–gravidous (>4 pregnancies) women, unlikely to reside in Ulanga district, and have no between–partner communication about family planning. Knowledge of these predictive factors is vital for intervention programs seeking to reduce unintended pregnancy within the study area. Vulnerable groups such as women who are young, unmarried, and the multi–gravidous should be targeted with interventions in order to enable them to avoid unintended pregnancies.

## Abbreviations

CI: Confidence interval; CRT: Cluster randomized trial; IRB: Institutional review board; KDHS: Kenya demographic and health survey; MDG: Millennium development goals; MRCC: Medical research coordinating committee; NIMR: National Institute for Medical Research; RRR: Relative risk ratio; TDHS: Tanzania demographic and health survey.

## Competing interests

The authors declare that they have no competing interests.

## Authors’ contributions

AE conceptualized the problem, designed the study, analyzed the data and drafted and revised various drafts of the manuscript. AMK provided assistance with data analysis, study design, and reviewed the manuscript critically. MN, KT, and HV reviewed the manuscript critically. AH and JFP developed a protocol for the Connect Project and reviewed this manuscript critically. All authors read and approved the final version of the manuscript.
